# A Novel *PAX6* Frameshift Mutation Identified in a Large Chinese Family with Congenital Aniridia

**DOI:** 10.3390/jpm13030442

**Published:** 2023-02-28

**Authors:** Chenghu Wang, Weihua Yang, Xiumiao Li, Chenchen Zhou, Jinghua Liu, Ling Jin, Qin Jiang, Yun Wang

**Affiliations:** 1 Eye Hospital, Nanjing Medical University, Nanjing 210029, China; 2 Shenzhen Eye Hospital, Shenzhen Eye Institute, Jinan University, Shenzhen 518040, China

**Keywords:** aniridia, *PAX6* gene, targeted gene capture sequencing, frameshift, autosomal recessive inheritance

## Abstract

Congenital aniridia is a rare autosomal dominant congenital ocular disorder. Genetic studies suggest that heterozygous mutations in the developmental regulator *PAX6* gene or the related regulatory regions leading to haploinsufficiency are the main cause of congenital aniridia. In this study, the clinical characteristics and pathogenic mutation of a four-generation Chinese family with congenital aniridia were investigated. All members recruited in this study underwent comprehensive ophthalmic examinations. Targeted gene capture sequencing and Sanger sequencing were performed to screen and confirm the candidate pathogenicity gene and its mutation. A multiple alignment of homologous sequences covering the identified mutation from different species was investigated, and the mutant protein structure was predicted using Swiss-Model. Additionally, the prediction of pathogenicity was analyzed using the ACMG Guidelines. Thirteen patients in this pedigree were diagnosed with congenital aniridia. A novel heterozygous frameshift mutation (c.391_398dupATACCAAG, p.Ser133Argfs*8) in exon 7 of the *PAX6* gene was identified in all affected individuals in the family. This study demonstrates that this frameshift mutation of the *PAX6* gene might be the causative genetic defect of congenital aniridia in this family. This mutation is predicted to cause the premature truncation of the PAX6 protein, leading to the functional haploinsufficiency of PAX6, which may be the major molecular mechanism underlying the aniridia phenotype. To the best of our knowledge, this is the first report of a novel pathogenic *PAX6* gene variant c.391_398dupATACCAAG(p.Ser133Argfs*8) identified in a Chinese family with congenital aniridia.

## 1. Introduction

Congenital aniridia (MIM 106210) is a rare panocular malformation with an incidence ranging from 1:64,000 to 1:100,000. It is characterized by either an absence or hypoplasia of the iris in both eyes [1]. It usually occurs with other ocular abnormalities such as cataracts, aniridia-related keratopathy, glaucoma, nystagmus, foveal hypoplasia, and obvious visual impairment. Congenital aniridia manifests in different forms, most of which are transmitted in an autosomal-dominant manner with a high degree of penetrance. It can also be sporadic, and as part of several syndromes including WAGR and WAGRO syndromes, as well as other syndromes with an intellectual impairment [2]. Studies have showed that about two-thirds of aniridia cases are familial, and the remaining one-third are sporadic [3]. The majority of congenital aniridia and other ocular disorders such as Peters anomaly occur due to different mutations found in or around the transcription factor *PAX6* gene (paired box 6, MIM 607108). The mutations reported in this gene are scattered throughout the complete coding sequence of the gene or in the regulatory regions. The mutations in the *PAX6* gene cause ocular abnormalities including aniridia in both vertebrate and invertebrate animal species. Studies have reported that the majority of *PAX6* mutations are heterozygous, resulting in the loss of one allele causing *PAX6* haploinsufficiency [4].

The *PAX6* gene is highly conserved throughout biological functions across diverse species, implicating its key role in embryonal ocular differentiation. It encodes a transcription factor, which is a 2.7 kb mRNA encoding a 422-amino-acid protein. It contains two DNA-binding domains: the paired domain (PD) and the homeodomain (HD). They are isolated by a linker segment (LNK), followed by a C-terminal region, rich in proline, serine, and threonine (PST) [5,6,7], which is a transactivation domain.

Numerous researchers have proven that the PAX6 protein is crucial for the normal development and maintenance of the eye, central nervous system, and many other elements [8,9,10,11]. Notably, *PAX6* is identified as the master gene controlling eye development. It is expressed in most ocular structures and plays key roles in lens induction, epithelial tissue morphogenesis, and neuronal specification or differentiation [12]. Mutations in the *PAX6* gene cause a series of ocular diseases, such as nystagmus, cataracts, and aniridia [13]. In recent years, many studies have focused on the varied clinical manifestation and allelic heterogeneity of *PAX6*-associated aniridia. In this study, a clinical and genetic evaluation of a four-generation Chinese family with congenital aniridia was carried out. A novel heterozygous frameshift mutation of the *PAX6* gene was identified. The molecular understanding of the predicted impact based on existing research and prediction algorithms was clarified. Moreover, the available clinical features of *PAX6*-associated aniridia in this study were investigated. To the best of our knowledge, this is the first reported association of the *PAX6* gene variant c.391_398dupATACCAAG, p.Ser133Argfs*8 with congenital aniridia.

## 2. Materials and Methods

### 2.1. Human Subjects

**Figure 1:**
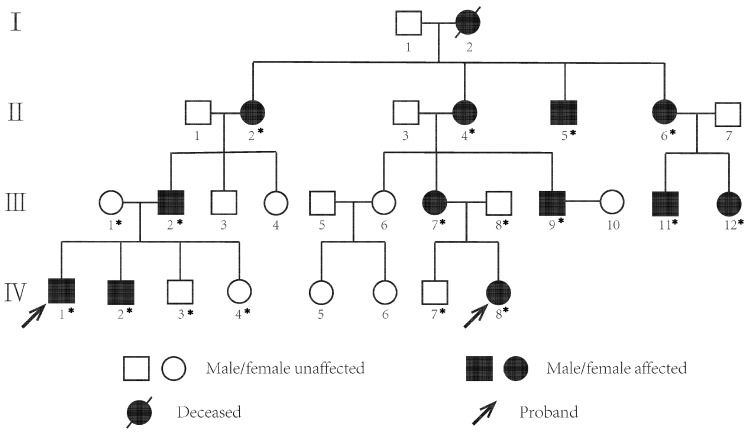
Pedigree of a Chinese family with congenital aniridia. Filled squares and circles denote affected males and females, respectively. Normal individuals are shown as empty symbols. The proband is indicated by an arrow. DNA samples used for sanger sequencing are marked with an asterisk.

A four-consecutive-generation Han Chinese pedigree (Figure 1) with congenital aniridia was recruited from The Affiliated Eye Hospital of Nanjing Medical University for the study. This family contained 29 individuals including 13 patients and 16 unaffected individuals. All procedures performed in studies involving human participants were in accordance with the ethical standards of the institutional and/or national research committee and with the 1964 Helsinki Declaration and its later amendments or comparable ethical standards. The study was approved by the Medical Ethics Committee of Eye Hospital, Nanjing Medical University (No. 2019004). Informed written consent was obtained from all participants prior to inclusion in the study.

### 2.2. Clinical Evaluation

All members recruited in this study underwent thorough ophthalmic examinations, including uncorrected and best-corrected visual acuity (BCVA), slit-lamp biomicroscopy of the anterior segment, eye position, eyeball movement, fundus examination after dilation, intraocular pressure (IOP) measurement, and optical coherence tomography.

### 2.3. Genomic DNA Extraction and DNA Library Preparation

After informed consent, blood samples were collected from all unrelated aniridia patients and most unaffected family members for DNA extraction and genomic analysis. The genomic DNA of all individuals was extracted from 2 mL of peripheral venous blood using a Blood Genomic DNA Extraction Kit (CoWin Bioscience, Beijing, China) according to the manufacturer’s instructions. DNA integrity was evaluated via 1% agarose gel electrophoresis. Targeted gene enrichment and sequencing were performed on the proband IV8. Genomic DNA was fragmented to an average size of 180 bp with sonication. Paired-end sequencing library preparation, comprising end repair, adapter ligation, and PCR enrichment, were carried out as recommended by Illumina protocols using DNA sample prep reagent set 1 (New England Biolabs (Beijing) LTD., Beijing, China).

### 2.4. Targeted Gene Enrichment and Sequencing

Targeted next-generation sequencing (NGS) was carried out with DNA probes designed to tile along the exon regions of 815 known pathogenic genes of hereditary ophthalmic disorders (Table S1), and the amplified DNA was captured using a GenCap capture kit (MyGenostics, Beijing, China) depending on the manufacturer’s instructions. The PCR product was purified by SPRI beads (Beckman Instruments, Brea, CA, USA) according to the manufacturer’s protocol. The enrichment libraries were sequenced on an Illumina HiSeq X ten sequencer (Illumina, San Diego, CA, USA) for 150 bp paired reads. Mutations were called after sequencing using BWA (http://bio-bwa.sourceforge.net/, accessed on 25 February 2023) and GATK (https://gatk.broadinstitute.org/hc/en-us, accessed on 25 February 2023) and annotated using ANNOVAR (http://annovar.openbioinformatics.org/en/latest/, accessed on 25 February 2023). The filtering process included all coding variants with an MAF < 5% and excluded synonymous and in-frame insertion/deletion variants. The identified mutation was named according to the nomenclature established by the Human Genomic Variation Society (HGVS). Candidate genes and variants were analyzed in combination with the patients’ phenotypes and public variant databases. Pathogenic genes and genetic variations with known, definitive genetic associations with aniridia were paid more attention, including *PAX6*, *ABCB6*, *FOXC1*, *PITX2*, *FOXD3*, and *CYP1B1*.

### 2.5. Sanger Sequencing

Direct Sanger sequencing was performed to determine the co-segregation of identified variants with the clinical phenotype in all affected family members and some normal members. The primer flanking region on exon 7 of the *PAX6* gene that covers the mutation Primers (5′–TGAAAGTATCATCATATTTGTAG–3′ (F) and 5′–AGGAGAGAGCATTGGGCTTA–3′ (R)) were designed using Primer Premier 5 and synthesized by BGI (Guangzhou, China). Polymerase chain reaction (PCR) was performed using a MyCycler thermal cycler (Bio-Rad, Hercules, CA, USA) in a 25 µL reaction system, which contained 0.1 µg of genomic DNA, 40 μmol/L forward and reverse primers, 3 mmol/L magnesiumchloride, and 2× Taq Master Mix (SinoBio, Shanghai, China). The PCR conditions used were as follows: 4 min at 95 °C for initial denaturation, 35 cycles of denaturation at 95 °C for 10 s for melting, annealing temperature of 54 °C lasting for 30 s, 30 s at 72 °C for extension, and a final additional extension step of 5 min at 72 °C. Before sequencing, 1% agarose gel electrophoresis was used to purify the target PCR fragments using the QIAquick Gel Extraction Kit (QIAGEN, Shanghai, China). Sanger sequencing was performed on a 3130XL sequencer and analyzed on an ABI 3130 Genetic Analyzer (Applied Biosystems, Thermo Fisher Scientific, Waltham, MA, USA). Sequence data were compared in a pairwise manner with the related human genome database (Assembly GRCh37/hg19).

### 2.6. Variant Analyses 

To analyze the evolutionary conservation of the mutant region, the sequences of *PAX6* orthologs in different vertebrate species were retrieved from the NCBI Reference Sequence database. A multiple alignment of homologous sequences from eight species was conducted using ClustalW2 (http://www.ebi.ac.uk/Tools/msa/clustalw2/, accessed on 25 February 2023). ACMG Guidelines were used through various algorithms for prediction of the impact of the variant on protein structure and the prediction of pathogenicity. Additionally, protein three-dimensional structures of full-length and mutated *PAX6* were evaluated with the Swiss-Model program (https://swissmodel.expasy.org/interactive, accessed on 25 February 2023).

## 3. Results

### 3.1. Clinical Features of the Family with Congenital Aniridia

**Figure 2:**
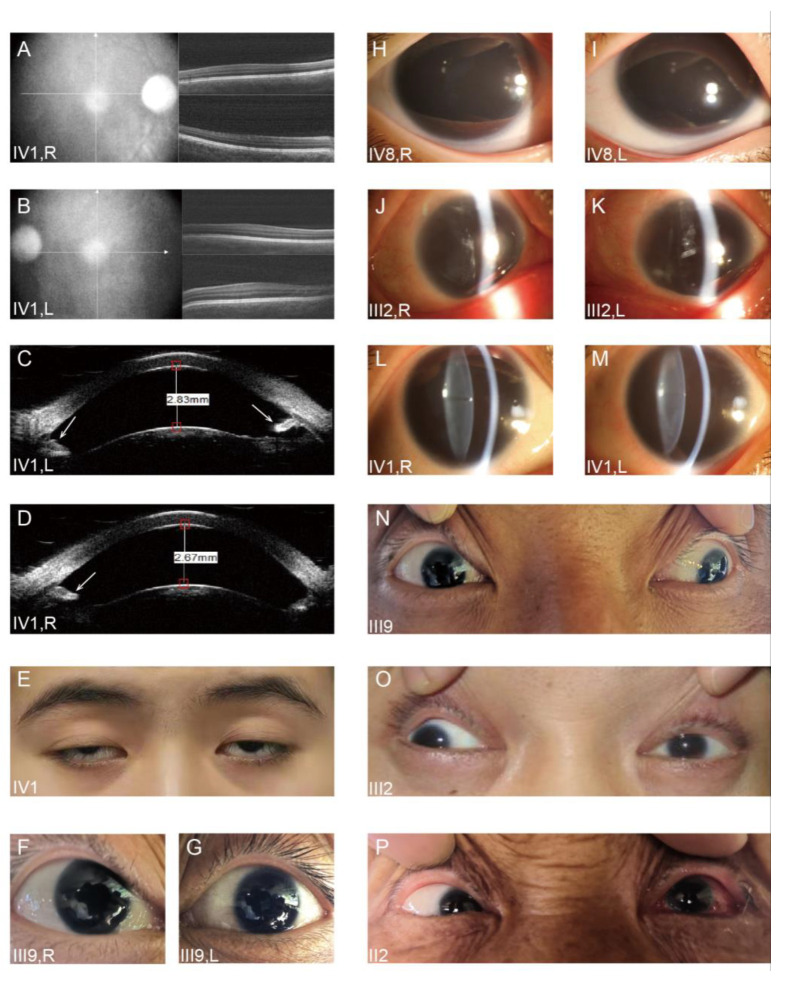
Representative photos of partial cases. (**A**,**B**) Optical coherence tomography images from the proband (IV1) demonstrated foveal hypoplasia in both the right eye (**A**) and the left eye (**B**). (**C**,**D**) Ultrasound biomicroscope photos displayed the iris remnants (the arrow) in the anterior chamber of the proband (IV1) in both eyes. (**E**) Ptosis was present in the proband (IV1). (**F**,**G**) Photos revealed total iris hypoplasia in both eyes of patient III9. (**H**,**I**) Anterior segment photographs indicated that both eyes of the proband IV8 exhibited complete iris absence. (**J**,**K**) The eyes of patient III2 were found with peripheral corneal edema and opacity, total iris hypoplasia, and aphakia by anterior segment photography. (**L**,**M**) Bilateral aniridia and cataract were observed in the proband (IV1). (**N**,**O**,**P**) Eye position photographs manifested that patient III9 (**N**) and patient III2 (**O**) suffered from exotropia, while patient II2 (**P**) developed esotropia.

The four-generation Chinese family enrolled in this study consisted of 29 individuals (Figure 1) with 13 patients diagnosed as congenital aniridia (I2 died). The probands IV1 and IV8 came to the hospital for treatment much earlier. Among all thirteen patients, seven (53.8%) were female, while the remaining six (46.2%) were male, with their ages varying from 3 to 69. All the affected members exhibited almost similar ocular symptoms, including complete absence of the irises, horizontal tremor, foveal hypoplasia, and uncorrected visual impairment in both eyes. Interestingly, peripheral corneal edema and opacification was observed in seven relatively older patients (53.8%), while lens opacity was also present in seven of thirteen patients (53.8%), indicating a positive correlation with age. In this respect, patient III2 deserved attention due to the experience of bilateral cataract surgery. Four of thirteen affected individuals (30.8%) suffered from strabismus, two (III2, III9) of thirteen patients (15.4%) suffered from exotropia, and the remaining two (II2, IV8) of thirteen patients (15.4%) suffered from esotropia. Except for proband IV1 who complained of ptosis, no other ocular or systemic abnormalities were detected. Ophthalmic manifestations of all patients and representative examination results are shown in Figure 2 and Table 1.

### 3.2. Molecular Analysis

**Figure 3:**
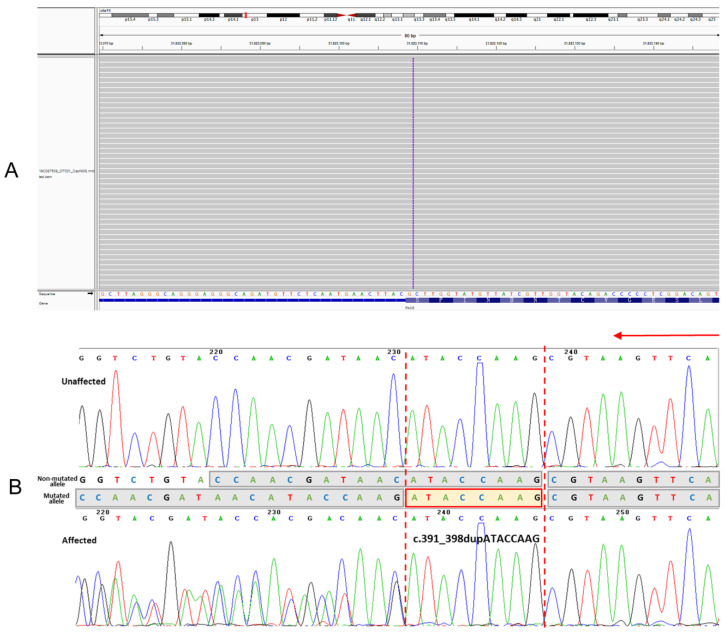
Sequencing results for the variant c.391_398dupATACCAAG of the *PAX6* gene. (**A**) Integrative genomics viewer visualization of whole-exome sequencing showing a novel heterozygous variant in the *PAX6* gene in the proband (IV8). (**B**) Sanger sequencing results of the *PAX6* gene confirmed the mutant allele with ATACCAAG duplicated at the position indicating by two red dotted line, in the affected individuals represented in the lower panel versus the normal individuals in the upper panel. Sequencing direction is indicated by an red arrow.

NGS was implemented for proband IV8. Detailed information about the sequencing and alignment quality of NGS data is provided in Table S2. Following NGS and the data analysis, a heterozygous frameshift mutation of the *PAX6* gene (NM_001604) was detected in proband IV8. *PAX6* was the most likely pathogenic gene in this study, which is further elaborated on in the discussion. This mutation (c.391_398dupATACCAAG, p.Ser133Argfs*8) was neither present in the databases of 1000 genome, ESP6500, dbSNP, EXAC, and HGMD, nor reported previously. To verify the identified gene defects, PCR and Sanger sequencing were applied to DNA samples, including 12 affected individuals and 5 unaffected family members who were available (Figure S1). The results manifested that all individuals with congenital aniridia of the pedigree harbored this heterozygous mutation. Diametrically, unaffected family members did not carry the variant (Figure 3). In brief, this mutation was co-segregated with the disease phenotype with complete penetrance.

**Figure 4:**
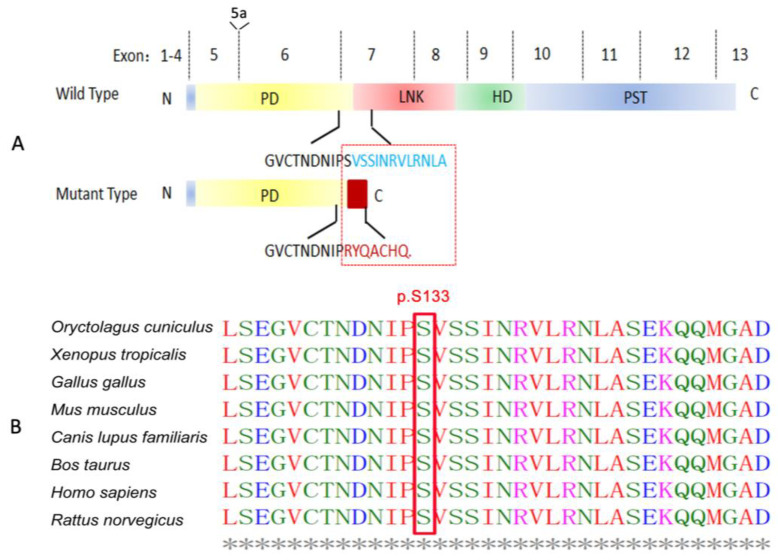
Schematic of predicted mutant PAX6 protein and bioinformatics analysis following ClustalW alignment. (**A**) Schematic of mutant PAX6 protein (lower panel) as predicted in this study compared with the wildtype PAX6 protein (upper panel). N, N terminus; PD, paired domain; LNK, linker domain; HD, homeodomain; PST, proline, serine, and threonine-rich domain; C, C terminus. Due to the frameshift duplication, the peptide in blue encoded by exon 7 of the wildtype is replaced by the peptide in red in the mutant in the box. (**B**) The *PAX6* mutation c.391_398dupATACCAAG involves a highly conserved residue. The serine at position 133 is highly conserved for PAX6, as demonstrated by an analysis of orthologs from eight different species: rabbit, tropical clawed frog, chicken, house mouse, dog, cattle, human, and Norway rat.

The *PAX6* gene variant (c.391_398dupATACCAAG, p.Ser133Argfs*8) was located in exon 7. This 8-bp duplication converts serine to arginine at amino-acid position 133, followed by another seven erroneous residues, and was predicted to create a truncated protein with 125 amino acids (Figure 4B) in comparison with a full-length wildtype protein of *PAX6* with 436 amino acids (Figure 4A). According to ACMG/AMP Guidelines for genomic variant classification, this variation was assessed for “pathogenic” with evidence of pathogenicity: PVS1, PM2, PP1, and PP4 (Table 2).

**Figure 5:**
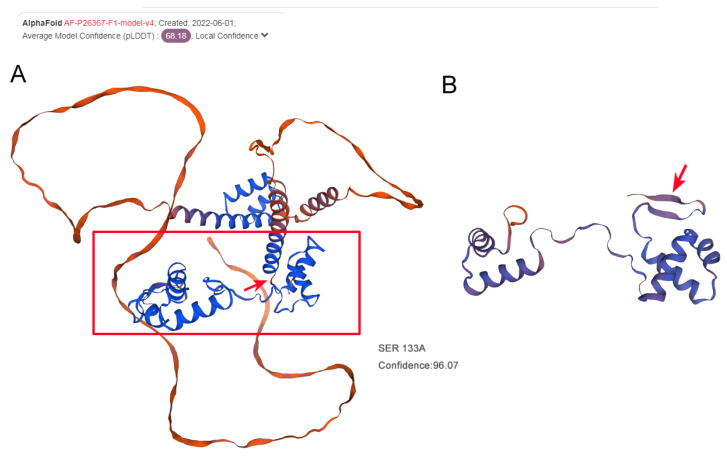
Three-dimensional structure prediction of wildtype PAX6 and mutated PAX6 protein. (**A**) Wildtype PAX6 structure. (**B**) Mutated PAX6 structure. The red arrow indicates the mutated position S133.

It was demonstrated that the serine at position 133 of PAX6 was highly conserved during evolution using ClustalW2 with multiple alignments of orthologs from eight different species: rabbit, tropical clawed frog, chicken, house mouse, dog, cattle, human, and Norway rat (Figure 4B). Bioinformatics analysis showed that the duplication of ATACCAAG at position c.391_398 may disrupt the open reading frames of *PAX6*, possibly retaining most of the paired domain of PAX6 but lacking the remaining domains, including the linker segment, the homeodomain, and the PST domain. Like other *PAX6* frameshift variants reported, this very likely causes haploinsufficiency due to nonsense-mediated decay (NMD). Even if the mutant transcript escapes NMD, the protein is expected to be nonfunctional. Swiss-Model predicted the 3D structure of PAX6 from the N terminal. The results indicated that mutated PAX6 even loses the α-helix of the paired domain (Figure 5), which may not be expected to be produced. The truncated protein might be unable to perform its substantial biological functions in ocular and neurologic development. Taken together, this variant of *PAX6* is a novel mutation which was most likely responsible for autosomal dominant congenital aniridia in this study.

## 4. Discussion

The *PAX6* gene, considered a causative gene for congenital aniridia since 1991 [6], is located at chromosome 11p13 in the assembly GRCh37/hg19, consists of 14 exons and 13 introns, and encodes 3 isoforms of transcripts determined by alternative splicing, including 1 isoform with an alternative exon between exon 5 and 6, called exon 5a [14]. The translation initiation codon is in exon 4, and the termination codon is in exon 14, with the first 3 being noncoding (Figure 5A). The alternative exonic inclusion of exon 5a generates a larger protein isoform PAX6-5a of 436 amino acids (NM_001604). It is highly conserved throughout biological evolution across different species, encoding a transcription factor which can initiate and regulate the transcription of downstream genes during embryogenesis via attaching to special areas of DNA [15]. PAX6 is expressed in almost all ocular structures including the iris, macula, optic nerve head, lens, and cornea [2,7], playing a key role in early eye development. PAX6 regulates the tissue-specific expression of different molecules, hormones, and structural proteins, and the transcription of target genes can only be initiated when the expression of PAX6 reaches a certain dose [16]. Mutations in the *PAX6* gene cause anterior segment malformations including aniridia, accompanied by a range of ocular phenotypes such as keratitis, cataract, glaucoma, nystagmus, foveal hypoplasia, and optic nerve disorders.

In this study, a novel heterozygous frameshift mutation of the *PAX6* gene (c.391_398dupATACCAAG, p.Ser133Argfs*8) was identified in a Chinese family. The genomic defects were present in all affected members but absent in unaffected family members, co-segregating with congenital aniridia, which is consistent with the previously reported pedigree. A small duplication of an ATACCAAG sequence at nucleotide position 391 of PAX6 was predicted to cause the replacement of a negatively charged serine by a positively charged arginine and generating or forming a premature stop signal eight codons downstream in exon 7. This altered the original open reading frame, which was expected to truncate PAX6 protein within the linker region. The resulting polypeptide then possibly retained the paired domain but lacked the homeodomain, the PST domain, and almost the entire linker segment. To our knowledge, outside of the homeodomain which combines with DNA directly, the PST domain is indispensable by acting as a strong activator, according to a single report which compared the transcriptional activation between wildtype and mutant PAX6 PST domains [7]. Researchers studied the attachment of wildtype or mutant PAX6 protein (TGA306 or TGA353) to the DNA-binding site of yeast GAL4, and then combined them with a plasmid containing the chloramphenicol acetyltransferase (CAT) gene. It was revealed that one mutant fusion protein (TGA353) stimulated CAT expression at only 5–10% of the wildtype level, whereas the other had no detectable CAT activity. Without testing, the novel mutation we described in this study truncated the PAX6 protein further upstream than what was examined in the mentioned article. Hence, it is highly possible that it eliminated the transcription activity. Although the DNA interaction is unlikely to be totally abolished as the paired domains are still there for DNA binding, we believe that the mutation has a non-negligible, negative affect, even deteriorating the function of remaining paired domains. Swiss-Model, sufficient for three-dimensional structure prediction, gave evidence which supports our conjecture [17,18,19,20]. It showed an apparent α-helix deletion (Figure 4), which contributes to the malformed PAX6 protein space structure and may perturb the affinity and specificity of DNA interaction, resulting in a decrease in protein function. On a gross level, the findings elucidated above may partially explain the molecular mechanisms of congenital aniridia.

Previous studies showed a wide spectrum of clinical manifestations accompanying aniridia, such as cataract, keratopathy, glaucoma, nystagmus, foveal hypoplasia, and low vision acuity [2,21]. Noticeably, phenotypes are severe when crossing onto an insertion/duplication mutation background [22]. Consistent with this, all mutation carriers in the family we recruited developed a complete loss of the iris, foveal hypoplasia, horizontal tremor, and vision impairment. A particularly compelling finding is that patients with the same mutation can exhibit various aniridia phenotypes, even in the same family. Available data of genotype and phenotype allowed for an exhaustive investigation in the pedigree we observed. Some associated diseases occurred in some, but not all individuals. Only four patients exhibited strabismus, while another one was diagnosed with ptosis. Among the members who suffered from corneal and/or lens opacity, the damage deteriorated with age. Regarding this phenomenon, we inferred that it may have derived from the haploinsufficiency of PAX6. Although many of these clinical features are present at birth, some can develop and/or progress later in life [11]. In addition to its activity in the embryo, PAX6 is maintained in specific cell types of the adult eye, including the corneal limbus, the iris, the pigmented ciliary body, the lens epithelium, and the Muller glia. This demonstrates that PAX6 activity may be implicated in adult self-renewal and regeneration of ocular structures, considering the functions of these cells [12]. Another alternative interpretation would be the NMD process. This pathway degrades some transcripts, bearing a premature translational termination codon, which aggravates or counteracts the effects of disease mutations [23]. These findings may partly reflect genetic heterogeneity; however, fully understanding the molecular mechanism remains a formidable challenge.

Until now, 550 mutations of the *PAX6* gene have been featured in the public version of the HGMD (http://www.hgmd.cf.ac.uk/ac/gene.php?gene=PAX6, accessed on 25 February 2023). These mutations are mainly devoted to aniridia, Peters abnormalities, foveal hypoplasia, nystagmus, cataracts, optic nerve dysplasia, and other eye diseases [13]. Roughly 30.0% are missense and nonsense mutations, while 11.6% are splicing site mutations. Small fragment deletions and insertions account for 23.6% and 10.5%, respectively. The remaining 20.2% of mutations are regulatory, gross duplication, complex rearrangements and other rare mutation types. Mutation types including nonsense and frameshift (insertions, duplication or deletions) will introduce a PTC and a consequent termination of translation, which are most commonly found in *PAX6*. In the present study, duplication mutation c.391_398dupATACCAAG of the *PAX6* gene results in a replacement of the subsequent amino-acid residues from position p.S133 with a peptide of eight erroneous amino-acid residues, including a PTC at the end. Singh and his colleagues demonstrated a dominant-negative mechanism using transient transfection assays with a variety of mutant PAX6 proteins featuring the C terminus half-truncated, co-expressed with wildtype PAX6 protein. All these mutant proteins lose most of the transactivation domain (PST), thereby acting as a repressor with no transactivation activity, but still maintaining DNA-binding domains (PD and HD) [24].

## 5. Conclusions

In summary, we analyzed the ocular phenotypes of 13 aniridia patients and identified a novel *PAX6* frameshift mutation as the causative gene defect in a four-generation Chinese family. This is a novel pathogenic mutation related to aniridia. Although the definite mechanism underlying how this variant in exon 7 of the *PAX6* gene triggers congenital aniridia is not yet clear, our study contributes additional information for future research. It expands the mutation spectrum of *PAX6*-related congenital aniridia, which is beneficial for prenatal diagnosis, genetic counseling, and gene therapy for familial cases in the near future. In addition, the molecular mechanism of genotype–phenotype correlations needs to be further investigated.

## Figures and Tables

**Table 1:** Clinical details of patients of the recruited family.

Pedigree Number	Gender	Age (Years)	BCVA (OD/OS)	IOP (mmHg) (OD/OS)	Aniridia	KP	Cataract	Macular Foveal Reflect	Nystagmus	Strabismus
II2	F	69	CF (20 cm)/0.12	21/19	Total	Corneal edema and opacity	+	−	+	Esotropia
II4	F	63	0.15/0.04	18/22	Total	Corneal edema and opacity	+	−	+	−
II5	M	59	0.02/CF (30 cm)	18/16	Total	Corneal edema and opacity	+	−	+	−
II6	F	50	0.06/CF (30 cm)	17/19	Total	Corneal edema and opacity	+	−	+	−
III2	M	46	0.01/CF (20 cm)	15/16	Total	Corneal edema and opacity	Aphakia	−	+	Exotropia
III7	F	34	0.12/0.1	20/21	Total	Corneal edema and opacity	+	−	+	−
III9	M	30	0.1/0.2	19/18	Total	Corneal edema and opacity	−	−	+	Exotropia
III11	M	24	0.3/0.3	17/20	Total	−	+	−	+	−
III12	F	20	0.06/0.1	18/19	Total	−	−	−	+	−
IV1	M	19	0.2/0.2	20/18	Total	−	+	−	+	−
IV2	M	3	0.1/0.3	16/17	Total	−	−	−	+	−
IV8	F	6	0.1-/0.1	19/19	Total	−	−	−	+	Esotropia

Abbreviations: M: male; F: female; BCVA: best-corrected visual acuity; CF: count fingers; OD: oculus dexter; OS: oculus sinister.

**Table 2:** Summary of the *PAX6* gene and its variant.

Gene	Position	Transcript	Exon	Change of Nucleotide	Predicted Change of Amino Acid	Domain	Variant Type	Status	ACMG/AMP Variant Classification
*PAX6*	chr11:31823109 *	NM_001604	7	c.391_398dupATACCAAG	p.Ser133Argfs*8	PD	Frameshift	Het	Pathogenic #

* Assembly GRCh37/hg19. # Evidence of pathogenicity: PVS1 + PM2 + PP1 + PP4. Abbreviations: Het, heterozygous; PD: paired domain.

## Data Availability

The data presented in this study are available on request from the corresponding author.

## Supplementary Materials

The following supporting information can be downloaded at: https://www.mdpi.com/article/10.3390/jpm13030442/s1, Table S1. Known pathogenic gene list of hereditary ophthalmic disorders; Table S2. Detail information about NGS data of proband IV8. Figure S1. Sanger sequencing results of *PAX6* gene on DNA samples from affected individuals and most family members who were available.
