# Supplementary material for: A Novel PAX6 Frameshift Mutation Identified in a Large Chinese Family with Congenital Aniridia
Source: J Pers Med. 2023 Feb 28;13(3):442. doi: 10.3390/jpm13030442 (PMC10052173; doi:10.3390/jpm13030442)
Supplement: Supplementary file 1 [file jpm-13-00442-s001.zip › Table S2.pdf]

Table S2. Detail information about NGS data of proband IV8.

Detail information about NGS data	Value or ratio
Raw data bases(Mb)	5144.76
Clean data bases(Mb)	5066.03
Aligned bases (Mb)	5057.47
Aligned	99.83%
Initial bases on target	2680649
Base covered on target	2678606
Coverage of target region	99.92%
Effective bases on target	1491661469
Fraction of effective bases on target	29.49%
Average sequencing depth on target	556.46
Fraction of target covered with at least 4X	99.88%
Fraction of target covered with at least 10X	99.80%
Fraction of target covered with at least 20X	99.63%
Duplication rate	22.94%
