# Supplementary figures and images for: A Novel PAX6 Frameshift Mutation Identified in a Large Chinese Family with Congenital Aniridia
Source: J Pers Med. 2023 Feb 28;13(3):442. doi: 10.3390/jpm13030442 (PMC10052173; doi:10.3390/jpm13030442)

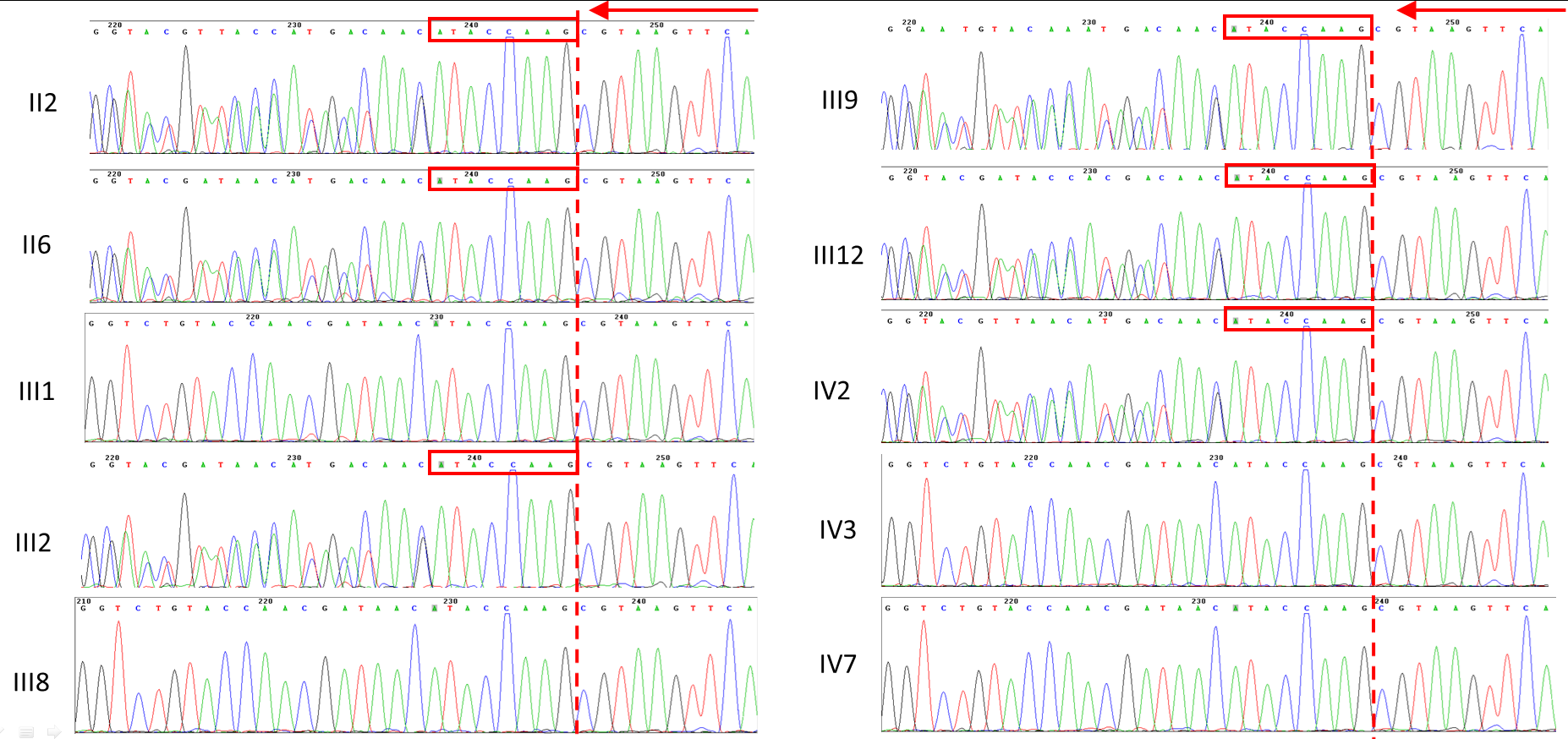

Supplement: Supplementary file 1 [file jpm-13-00442-s001.zip › Figure S1.tif]
